# Bedside Renal, Inferior Vena Cava, Pulmonary, Pleural, Epicardial, Diaphragm (BRIPPED) Scan for Evaluating Patients Presenting With Dyspnea in the Emergency Department

**DOI:** 10.7759/cureus.104873

**Published:** 2026-03-08

**Authors:** Nilesh Jha, Krunalkumar Pancholi, Rina Parikh, Jigar Rathwa, Mayank Pandya

**Affiliations:** 1 Department of Emergency Medicine, Sir Sayajirao General (SSG) Hospital and Medical College, Vadodara, IND

**Keywords:** acute dyspnea, bripped scan, diagnostic accuracy, emergency department, multiorgan ultrasound, point-of-care ultrasound

## Abstract

Background: Acute dyspnea is a common and challenging presentation in the emergency department (ED), with multiple potential etiologies. Rapid and accurate bedside evaluation is critical to guide timely management. The Bedside Renal, Inferior Vena Cava, Pulmonary, Pleural, Epicardial, Diaphragm (BRIPPED) scan is a focused, multiorgan ultrasound protocol incorporating assessment of pulmonary B-lines, right ventricular size, inferior vena cava collapsibility, pleural and pericardial effusion, pneumothorax, left ventricular ejection fraction, and lower extremity deep venous thrombosis, designed to differentiate the causes of acute dyspnea in the ED.

Methods: We conducted a prospective observational study of 148 adult patients presenting to the ED with acute dyspnea. Each patient underwent a BRIPPED scan at the time of presentation. Scan findings were compared with the final diagnosis established through clinical evaluation, laboratory investigations, imaging, and specialist consultation. Diagnostic performance metrics, including sensitivity, specificity, positive predictive value (PPV), and negative predictive value (NPV), were calculated for cardiac, pulmonary, mixed (cardiac plus pulmonary), and noncardiopulmonary causes of dyspnea.

Results: For cardiac causes, the BRIPPED scan demonstrated a sensitivity of 98.46% and a specificity of 96.39%, with a PPV of 95.52% and an NPV of 98.77%. For pulmonary causes, sensitivity was 100%, specificity 95.65%, PPV 93.33%, and NPV 100%. In mixed etiologies, sensitivity and specificity were 20% and 100%, respectively, with a PPV of 100% and an NPV of 97.28%. For noncardiopulmonary causes of dyspnea, the protocol showed a sensitivity of 86.36%, a specificity of 99.21%, a PPV of 95.00%, and an NPV of 97.66%.

Conclusion: In this single-center study, the BRIPPED ultrasound protocol demonstrated high diagnostic accuracy for broad etiologic categorization of acute dyspnea. The protocol showed particularly strong performance for identifying cardiac and pulmonary causes. Its implementation may improve early diagnostic accuracy and facilitate timely patient management. Multicenter validation with blinded adjudication and a larger sample size is warranted to assess generalizability and reduce biases.

## Introduction

Acute dyspnea is one of the most common and challenging presentations in the emergency department (ED). Evaluation and management of acute dyspnea in the ED remain challenging for physicians. Diagnostic uncertainty is common at presentation and often leads to delays in diagnosis and initiation of appropriate treatment, which may adversely affect patient outcomes. Given the time-sensitive nature of many underlying conditions, there is a critical need for rapid and accurate diagnostic strategies to guide early management and improve prognosis [1,2]. According to the American Thoracic Society, dyspnea is defined as a “subjective experience of breathing discomfort.” As a symptom, dyspnea can only be perceived and reported by the individual experiencing it [3]. This subjectivity poses a significant challenge in emergency settings. Patients presenting with sudden-onset dyspnea are frequently anxious and distressed, which can worsen their perceived symptoms and further complicate history taking. In many cases, the clinical examination is also limited due to associated features such as confusion, loss of consciousness, cyanosis, inability to speak in full sentences, or respiratory exhaustion. These factors can significantly hinder effective clinical assessment by the treating physician [3]. Moreover, prolonged history taking or reliance on sequential investigations may consume critical time in unstable patients.

Dyspnea has a broad etiological spectrum, encompassing cardiac, pulmonary, mixed, and noncardiopulmonary causes. Identifying the underlying etiology in the emergency setting can be exhausting and challenging for the treating physician, particularly in undifferentiated presentations. In recent years, emergency ultrasound (EUS) has emerged as a valuable adjunct to traditional clinical assessment by providing rapid, bedside, real-time diagnostic information [4]. However, the optimal role of EUS in clinical decision-making and its integration into routine emergency practice continue to be areas of ongoing research. Several ultrasound-based diagnostic protocols have been developed for evaluating dyspnea, including the Bedside Lung Ultrasound in Emergency (BLUE) and Fluid Administration Limited by Lung Sonography protocols [5]. While these protocols have demonstrated utility in emergency settings, they primarily focus on pulmonary pathology and provide limited assessment of cardiac function, volume status, and thromboembolic disease.

The Bedside Renal, Inferior Vena Cava, Pulmonary, Pleural, Epicardial, Diaphragm (BRIPPED) protocol is a relatively newer, streamlined, multiorgan ultrasound approach that combines focused lung ultrasound, cardiac sonography, inferior vena cava (IVC) collapsibility assessment, and evaluation for deep vein thrombosis (DVT). Unlike other commonly used protocols, BRIPPED does not require patient repositioning into the supine posture and omits advanced echocardiographic techniques such as tissue Doppler imaging. These features make it particularly suitable for real-world application in busy EDs [6]. By incorporating both cardiac and pulmonary assessment along with DVT evaluation, the BRIPPED protocol offers a comprehensive bedside approach for patients presenting with undifferentiated dyspnea.

The primary objective of this study was to determine the diagnostic performance of the BRIPPED (Bedside Renal, Inferior Vena Cava, Pulmonary, Pleural, Epicardial, Diaphragm) ultrasound protocol in classifying undifferentiated dyspnea into broad categories, cardiac, pulmonary, mixed, or noncardiopulmonary, in patients presenting to the ED. Diagnostic performance was evaluated by comparing the provisional diagnosis based on the BRIPPED protocol with the final discharge diagnosis established after comprehensive clinical assessment and appropriate investigations. The primary outcome measures included sensitivity, specificity, positive predictive value (PPV), and negative predictive value (NPV), likelihood ratios (LRs), and overall diagnostic accuracy.

## Materials and methods

Study design

This study was a prospective, observational diagnostic accuracy study designed to assess the diagnostic performance of a multiorgan point-of-care ultrasound (POCUS) strategy using a predefined protocol known as the BRIPPED scan. The protocol was applied to evaluate patients presenting to the ED with acute undifferentiated dyspnea.

Study setting and population

The study was conducted between June 2024 and January 2025 in the ED of a tertiary care hospital in Western India, which has an annual ED census of approximately 50,000 patient visits. The ED serves as a primary teaching site for emergency medicine residents, where bedside ultrasound is routinely used in clinical practice under the supervision of faculty consultants.

Patients presenting to the ED with acute undifferentiated dyspnea were screened for eligibility. Undifferentiated dyspnea was defined as dyspnea in which the treating physician, at the time of initial clinical assessment, considered two or more possible etiologies. A total of 148 adult and adolescent patients aged 12 years or older were enrolled in the study. Patient recruitment was performed when the consultant investigator was present in the ED.

Exclusion criteria

The exclusion criteria included the following: 1) a final diagnosis could not be established due to death during evaluation or leave against medical advice, 2) the patient refused admission to the study institution, 3) dyspnea was secondary to trauma, and 4) the patient was referred with a previously established diagnosis.

The study protocol was approved by the Institutional Ethics Committee. Written informed consent was obtained from all patients or, when appropriate, from their legally authorized representatives or attendants.

Sample size

The sample size was calculated using the formula $n = \frac{Z^{2} \times P \times (1 - P)}{d^{2}}$, where P represents the anticipated sensitivity of the BRIPPED scan, Z corresponds to a 95% confidence level, and d denotes an absolute precision of 5%. The anticipated sensitivity was assumed to be 90%, based on findings from a pilot study conducted on a similar population [7]. The calculated sample size was 139 patients. After adding a 10% buffer to account for potential exclusions or technical limitations, the final target sample size was set at 153. Five patients were subsequently excluded due to technical issues during data entry, resulting in a final analyzed sample of 148 patients.

Study protocol

The BRIPPED ultrasound protocol is a multiorgan point-of-care ultrasound approach designed for the bedside evaluation of patients presenting with acute dyspnea in the ED. The protocol was originally described by Stewart et al., who performed a standardized ultrasound assessment of pulmonary B-lines, right ventricular (RV) size, IVC collapsibility, pleural and pericardial effusion, pneumothorax, left ventricular (LV) ejection fraction, and lower extremity deep venous thrombosis (DVT) in dyspneic patients presenting to the ED [6]. The primary objective of their study was to assess the impact of the BRIPPED scan on the treating physician’s differential diagnosis. They reported that the BRIPPED scan influenced physician diagnostic thinking to a degree comparable to laboratory and radiographic investigations but did not significantly alter the final diagnosis.

In the present study, the same ultrasound protocol was used; however, the objective differed. Our aim was to evaluate the diagnostic validity and accuracy of the BRIPPED protocol by comparing provisional diagnoses derived from the BRIPPED scan with final diagnoses established after complete clinical evaluation and relevant investigations.

All ultrasound examinations were performed at the bedside using standard ED ultrasound equipment. The BRIPPED protocol consists of the following components.

B: Pulmonary B-Lines

Pulmonary ultrasound was performed using a 6-13 MHz linear transducer. Lung scanning was conducted in accordance with the BLUE protocol. Anterolateral lung zones were examined routinely, and posterior lung zones were assessed with the patient in a sitting position or by gently turning the patient when feasible. Pulmonary B-lines were defined as the presence of three or more vertical, hyperechoic reverberation artifacts per intercostal space. These artifacts originated from the pleural line, extended to the bottom of the screen without fading, moved synchronously with lung sliding, and erased horizontal A-lines [6,8].

*Interpretation*: Diffuse, bilateral B-lines involving multiple lung zones were considered suggestive of pulmonary edema. Pulmonary edema was classified as cardiogenic when associated abnormalities were noted on focused cardiac ultrasound. The presence of predominant A-lines with preserved lung sliding and absence of B-lines was considered suggestive of asthma or chronic obstructive pulmonary disease (COPD) in patients with a compatible clinical history. Lower respiratory tract infection was suspected when lung ultrasound demonstrated localized pleural line irregularities, subpleural consolidations, tissue-like patterns, pleural shredding, or areas of reduced or absent lung sliding.

R: RV Size

RV size was assessed using the phased-array probe in the apical four-chamber view. RV strain was considered present when the RV appeared equal to or larger than the left ventricle on visual assessment or when the RV/LV ratio exceeded 0.9 using direct comparison methods [1,6,9].

*Interpretation*: Pulmonary embolism was suspected in patients with a compatible clinical history, absence of significant lung ultrasound findings, and evidence of RV strain.

I:* IVC Assessment*

The IVC was visualized using a subcostal longitudinal view with a 2-5 MHz curvilinear probe. IVC collapsibility was assessed during inspiration and categorized as reduced (<50%) or normal to increased (>50%) [1,6,9].

*Interpretation*: Reduced IVC collapsibility (<50%) was interpreted as suggestive of elevated right atrial pressure or intravascular volume overload, whereas complete or near-complete collapsibility was considered indicative of volume depletion.

P: Pleural and Pericardial Effusion

Pleural effusions were evaluated by scanning bilateral costophrenic angles using a linear probe. Pleural effusion was identified as an anechoic space between the parietal and visceral pleura, typically above the diaphragm, with occasional visualization of the thoracic spine sign.

Pericardial effusion was assessed in parasternal long-axis views and identified as an anechoic space between the visceral and parietal pericardium.

P: Pneumothorax

Evaluation for pneumothorax was performed using a linear probe along the anterior chest wall, particularly in the midclavicular line. Pneumothorax was diagnosed based on the absence of lung sliding, the presence of a barcode sign on M-mode, and, when visualized, the lung point.

E: LV Ejection Fraction

LV systolic function was assessed using parasternal long-axis and short-axis views with a phased-array probe. Ejection fraction was estimated visually and categorized as normal (>55%), mildly reduced (45%-55%), or markedly reduced (<45%) [6,9-11].

*Interpretation*: Congestive heart failure was diagnosed when a reduced or markedly reduced ejection fraction was observed in association with diffuse pulmonary B-lines. Acute coronary syndrome was suspected when regional wall motion abnormalities were identified.

D: Deep Venous Thrombosis

Compression ultrasonography of the femoral and popliteal veins was performed using a linear probe. DVT was diagnosed when a thrombus was directly visualized or when the vein failed to completely collapse under probe compression while the accompanying artery demonstrated compressibility [6,12,13].

*Interpretation*: The presence of DVT in conjunction with RV strain and reduced IVC collapsibility was considered supportive of a diagnosis of pulmonary embolism.

Following completion of the BRIPPED scan, a provisional diagnosis was made in the ED. For the purpose of analysis, provisional diagnoses were categorized as cardiac, pulmonary, mixed, or noncardiopulmonary causes of dyspnea as mentioned in Table 1. The same categorization was applied to the final diagnosis.

**Table 1:** Classification used for dyspnea in our study COPD: chronic obstructive pulmonary disease

Main category	Specific diagnoses
Pulmonary causes	Pneumonia, acute exacerbation of COPD, acute exacerbation of asthma, pneumothorax, pleural effusion, and acute respiratory distress syndrome
Cardiac causes	Congestive heart failure, acute coronary syndrome, pericardial effusion or cardiac tamponade, pulmonary embolism
Mixed causes	Any one of the cardiac causes along with any of the pulmonary cause like heart failure with concurrent COPD exacerbation, heart failure with lower respiratory tract infection
Other causes	Noncardiac and nonpulmonary causes (e.g., acute or chronic kidney injury with fluid overload, anxiety, or panic disorder)

The reference standard was defined as the final discharge diagnosis recorded in the official discharge summary and verified by the treating consultant physician. This diagnosis was established through thorough clinical assessment, supported by appropriate objective laboratory and imaging investigations, specialist input when necessary, and the patient’s clinical progression during hospitalization.

A mixed (cardiac and pulmonary) etiology was assigned when both conditions were objectively confirmed, deemed by the treating team to meaningfully contribute to the patient’s presentation, and documented as co-primary diagnoses at discharge. If one condition was considered secondary or incidental, the primary diagnosis was determined based on overall clinical judgment and supporting investigations.

Ultrasound operators and training

All BRIPPED scans were performed by emergency medicine residents (postgraduate years 2-3) and were credentialed by the department of emergency medicine and had at least six months of experience in performing POCUS examinations in the ED. Senior emergency medicine faculty with advanced POCUS certification were available for consultation in technically challenging cases.

Data entry and statistical analysis

Data were collected using a structured data collection form designed specifically for the study. The form was used to record patient demographics, presenting clinical features, vital signs at presentation, relevant laboratory investigations, BRIPPED ultrasound findings, and final diagnosis. All collected data were entered into a secure electronic database using Google Sheets (Google, Mountain View, CA) and were anonymized prior to analysis to ensure patient confidentiality. Sensitivity, specificity, PPV, NPV, positive LR (+LR)/negative LR (-LR) were computed for each etiology using 2 x 2 contingency tables. Data analysis was performed using Microsoft Excel (Microsoft Corporation, Redmond, WA). Associations between BRIPPED scan diagnosis and final discharge diagnosis were assessed using the chi-square test. Fisher’s exact test was used when the expected cell counts were fewer than five. A p value of less than 0.05 was considered statistically significant.

## Results

Demographic characteristics

A total of 148 patients presenting with acute undifferentiated dyspnea were included in the final analysis. The study population consisted predominantly of older adults, with 67 patients (45.3%) aged over 50 years, 51 patients (34.9%) aged between 30 and 50 years, and 30 patients (20.3%) aged between 12 and 30 years. The mean age of the participants was 48.2 years. There was a male predominance, with 98 male patients (66.2%) and 50 female patients (33.8%).

Clinical presentation and vital parameters

On initial assessment, the majority of patients presented with tachypnea, with 132 patients (89.0%) having a respiratory rate greater than 24 breaths/minute. Hypoxia (SpO₂ <90% on room air) was observed in 90 patients (60.8%). Tachycardia (heart rate >100 beats/minute) was documented in 63 patients (42.6%). Hypotension, defined as systolic blood pressure <90 mmHg, was present in 69 patients (46.6%), while hypertension (systolic blood pressure >160 mmHg) was noted in 32 patients (21.6%). Based on dyspnea severity assessment, 59 patients (39.9%) were classified as having grade 3 dyspnea and 34 patients (23.0%) as grade 4 dyspnea. Vital parameters are summarized in Table 2.

**Table 2:** Clinical examination of patients SpO_2_: peripheral oxygen saturation; SBP: systolic blood pressure

Parameter	Value
Tachycardia (heart rate >100 bpm)	42.5%
Hypoxia (SpO_2_ <90%)	60.8%
Hypotension (SBP <90 mmHg)	46.6%
Hypertension(SBP >160 mmHg)	21.6%
Dyspnea	
Grade 4	23%
Grade 3	39.9%
Tachypnea (respiratory rate >24 breaths/minute)	89%

Associated symptoms

Among the study population, the most commonly reported associated symptoms were cough (44.5%), fever (30%), chest pain (29%), and orthopnea or paroxysmal nocturnal dyspnea (23%). When stratified by final diagnosis, distinct symptom patterns were observed across different etiological categories. The distribution of associated symptoms among cardiac, pulmonary, mixed, and noncardiopulmonary causes is summarized in Table 3.

**Table 3:** Symptomatology of different causes of dyspnea PND: paroxysmal nocturnal dyspnea

Symptom	Cardiac	Respiratory	Mixed	Other
Cough	12 (18.18%)	49 (74%)	3 (4.5%)	2 (3%)
Fever	5 (11.3%)	32 (72.7%)	2 (4.5%)	5 (11.3%)
Chest pain	15 (34.8%)	21 (48.8%)	3 (7%)	4 (9.3%)
Orthopnea/PND	21 (60%)	12 (34.3%)	2 (5.7.0%)	0 (0.0%)
Palpitations	14 (60.8%)	08 (34.7%)	1 (4.3%)	0 (0.0%)
Anxiety	15 (31.2%)	17 (35.4%)	3 (6.2%)	13(27%)

Etiological distribution of dyspnea

Based on provisional diagnoses made using the BRIPPED protocol, cardiac etiology was identified in 67 patients (45.3%), respiratory etiology in 60 patients (40.5%), mixed etiology in one patient (0.7%), and noncardiopulmonary causes in 20 patients (13.5%). According to final discharge diagnoses, dyspnea was attributed to cardiac causes in 65 patients (43.8%), pulmonary causes in 56 patients (37.9%), mixed causes in five patients (3.4%), and noncardiopulmonary causes in 22 patients (14.9%). A comparison between BRIPPED-based provisional diagnoses and final diagnoses is presented in Table 4.

**Table 4:** Distribution of causes of dyspnea by BRIPPED scan and final diagnosis BRIPPED: Bedside Renal, Inferior Vena Cava, Pulmonary, Pleural, Epicardial, Diaphragm

Cause of dyspnea	Number of patients (n) with diagnosis made by BRIPPED	Number of patients (n) by final diagnosis
Cardiac etiology	67 (45.3%)	65 (43.8%)
Respiratory etiology	60 (40.5%)	56 (37.9%)
Mixed etiology	1 (0.68%)	5 (3.4%)
Other etiologies	20 (13.8%)	22 (14.9%)
Total	148	148

Diagnostic performance of the BRIPPED protocol

The diagnostic performance of the BRIPPED ultrasound protocol was evaluated by comparing provisional diagnoses with final discharge diagnoses. The overall diagnostic accuracy of the BRIPPED protocol was 97.3% with a Cohen’s kappa of >0.9.

For cardiac causes of dyspnea, the BRIPPED protocol demonstrated a sensitivity of 98.46% and a specificity of 96.39%. The PPV was 95.52%, and the NPV was 98.77%. The +LR was 27.35, and the -LR was 0.016 with a p value of <0.001 (chi-square test).

For respiratory causes, the protocol achieved a sensitivity of 100.0% and a specificity of 95.65%. The PPV was 93.33%, and the NPV was 100.0%. The +LR was 22.98, and the -LR was 0.0 with a p value of <0.001 (Fisher’s exact test).

For mixed etiologies, the sensitivity was 20.0%, with a specificity of 100.0%. The PPV was 100.0%, and the NPV was 97.28%. The -LR was 0.80. The association between BRIPPED diagnosis and final mixed diagnosis was statistically significant (Fisher’s exact test, p = 0.034).

For noncardiopulmonary causes, the BRIPPED protocol demonstrated a sensitivity of 86.36% and a specificity of 99.21%. The PPV was 95.00%, and the NPV was 97.66%. The +LR was 109.31, and the -LR was 0.137 with p value <0.001 (Fisher’s exact test). A summary of diagnostic performance metrics across the four etiological categories is presented in Table 5. The contingency tables used for calculating sensitivity, specificity, PPV, and NPV are provided in the Appendix.

**Table 5:** Overall sensitivity, specificity, positive predictive value, negative predictive value, positive likelihood ratio, and negative likelihood ratio of all the four divisions according to causes of dyspnea PPV: positive predictive value; NPV: negative predictive value; +LR: positive likelihood ratio; -LR: negative likelihood ratio

Etiology	n (%)	Sensitivity	Specificity	PPV	NPV	+LR	-LR	p value
Cardiac	65 (43.8)	98.46%	96.39%	95.52%	98.77%	27.35	0.016	<0.001
Respiratory	56 (37.9)	100.0%	95.65%	93.33%	100.0%	22.98	0.0	<0.001
Mixed	5 (3.4)	20.0%	100.0%	100.0%	97.28%	-	0.80	<0.05
Other	22 (14.9)	86.36%	99.21%	95.00%	97.66%	109.31	0.137	<0.001

## Discussion

In this prospective study of 148 patients presenting with acute dyspnea to a tertiary care ED, we evaluated the diagnostic performance of the BRIPPED POCUS protocol in identifying the underlying etiology of dyspnea. The study population predominantly comprised middle-aged and older adults, with a mean age of 48.2 years, reflecting the increasing burden of cardiorespiratory diseases in this age group in the Indian setting. Similar age distributions among dyspneic patients have been reported in previous Indian ED-based studies [14].

A male predominance was observed in our cohort. While Indian studies specifically demonstrating gender-based distribution of acute dyspnea in emergency settings are limited, this finding may be related to differences in exposure to cardiovascular and respiratory risk factors such as tobacco use, occupational hazards, and lifestyle factors, which are known to be more prevalent among Indian men. This observation is supported by population-based studies, including those by Prabhakaran et al., which have documented a higher prevalence of cardiovascular risk factors among Indian males [15].

The majority of patients in our study presented with significant physiological derangements, including tachypnea, hypoxia, tachycardia, and hypotension. Additionally, nearly two-thirds of patients were classified as New York Heart Association grade 3 or 4, indicating moderate-to-severe functional limitation. These findings suggest that patients often present to EDs at an advanced stage of illness. This pattern may reflect delayed health-seeking behavior or referral delays, which are frequently encountered in resource-limited settings, and have also been described in regional studies from neighboring countries such as Nepal [16].

With respect to etiological distribution, cardiac and pulmonary causes together accounted for the majority of dyspnea presentations in our cohort. Although few studies have categorized dyspnea into broad etiological groups similar to those used in our analysis, prior literature consistently reports that conditions such as lower respiratory tract infections, acute decompensated heart failure, COPD, and asthma constitute a substantial proportion of dyspnea-related emergency visits [17]. The distribution observed in our study is, therefore, broadly consistent with existing literature.

The BRIPPED ultrasound protocol demonstrated high diagnostic performance for both cardiac and pulmonary causes of dyspnea. Sensitivity and specificity values observed in our study are comparable to those reported in previous studies evaluating lung and cardiac ultrasound in emergency settings. Volpicelli et al. and the BLUE protocol have highlighted the high diagnostic accuracy of lung ultrasound in identifying common respiratory emergencies, including pulmonary edema, pneumonia, and pneumothorax [8,18]. Similarly, integrated cardiac ultrasound protocols have been shown to reliably identify heart failure in acutely dyspneic patients, as reported by Gargani et al. [19]. Pivetta et al. reported a pooled sensitivity and specificity of 94% and 92%, respectively, for ultrasound-based diagnosis of acute heart failure, findings consistent with our study [20].

The high NPV observed for both cardiac and pulmonary etiologies suggests that the BRIPPED protocol may be particularly useful in excluding major cardiopulmonary causes of dyspnea during initial ED evaluation. In high-volume and resource-limited emergency settings, such rapid exclusion of life-threatening conditions may assist clinicians in prioritizing further diagnostic testing and management, although outcome-based benefits were not specifically assessed in this study.

Diagnostic performance was comparatively lower in patients with mixed etiologies of dyspnea. This is not unexpected, as overlapping cardiac and pulmonary pathologies can result in shared ultrasound findings, such as the presence of B-lines in both acute decompensated heart failure and pulmonary parenchymal disease. In such cases, reliance on ultrasound findings alone may be insufficient, and integration with laboratory parameters, radiological imaging, and clinical judgment becomes essential. This highlights the role of the BRIPPED protocol as an adjunct rather than a standalone diagnostic tool, particularly in complex presentations [20,21].

For noncardiopulmonary causes of dyspnea, the BRIPPED protocol demonstrated high specificity and a favorable -LR. A normal BRIPPED scan substantially reduced the posttest probability of a primary cardiopulmonary etiology, thereby prompting clinicians to consider alternative diagnoses. This aspect of the protocol may help streamline diagnostic pathways by reducing unnecessary cardiopulmonary investigations in selected patients, though this requires further validation.

Limitations

This study has several limitations. It was conducted at a single tertiary care center, which may limit the generalizability of the findings. Ultrasound examinations were operator-dependent, and although scans were performed by trained emergency physicians, interobserver variability was not assessed. The study was not blinded, as treating physicians were aware of ultrasound findings during clinical decision-making. The number of patients with mixed etiologies was relatively small, which may have affected the diagnostic performance metrics in this subgroup.

## Conclusions

In this single-center prospective study, the BRIPPED POCUS protocol demonstrated high diagnostic performance for broad etiologic categorization of patients presenting with acute undifferentiated dyspnea. Performance was particularly strong for cardiac and pulmonary categories, while sensitivity was lower in the mixed-etiology subgroup. Based on these diagnostic accuracy findings, the BRIPPED protocol shows promise as a bedside tool for etiologic categorization of undifferentiated dyspnea. However, prospective studies evaluating time to reach diagnosis, clinical impact, workflow integration, resource utilization, and patient outcomes are needed to establish its implementation decisions. independent multicenter validation with blinded adjudication and larger mixed-etiology samples will be essential to provide less biased accuracy estimates, assess generalizability, and determine the protocol’s true clinical utility.

## Appendices

**Table 6:** 2 × 2 contingency table for performance of BRIPPED scan in cardiac causes BRIPPED: Bedside Renal, Inferior Vena Cava, Pulmonary, Pleural, Epicardial, Diaphragm; TP: true positive; FN: false negative

BRIPPED scan	Final diagnosis	
	Cardiac	Not cardiac
Cardiac	TP = 64	FP = 3
Not cardiac	FN = 1	TN = 80

**Table 7:** 2 × 2 table for performance of BRIPPED scan in respiratory causes BRIPPED: Bedside Renal, Inferior Vena Cava, Pulmonary, Pleural, Epicardial, Diaphragm; TP: true positive; FN: false negative

BRIPPED scan	Final diagnosis	
	Respiratory	Not respiratory
Respiratory	TP = 56	FP = 4
Not respiratory	FN = 0	TN = 88

**Table 8:** 2 × 2 table for performance of BRIPPED scan in mixed cardiac and respiratory causes BRIPPED: Bedside Renal, Inferior Vena Cava, Pulmonary, Pleural, Epicardial, Diaphragm; TP: true positive; FN: false negative

BRIPPED scan	Final diagnosis	
	Mixed cause	Not mixed
Mixed	TP = 1	FP = 0
Not mixed	FN = 4	TN = 143

**Table 9:** 2 × 2 table for performance of BRIPPED scan in other causes

BRIPPED scan	Final diagnosis	
	Other causes	Not others
Other	TP = 19	FP = 1
Not others	FN = 3	TN = 125
